# Antimicrobial and Antibiofilm Activity of *Origanum vulgare* Extracts Obtained by Supercritical Fluid Extraction Under Various Extraction Conditions

**DOI:** 10.3390/molecules29245823

**Published:** 2024-12-10

**Authors:** Daniela Gwiazdowska, Agnieszka Waśkiewicz, Krzysztof Juś, Katarzyna Marchwińska, Szymon Frąk, Dominik Popowski, Katarzyna Pawlak-Lemańska, Pascaline Aimee Uwineza, Romuald Gwiazdowski, Daria Padewska, Marek Roszko, Marcin Bryła

**Affiliations:** 1 Department of Natural Science and Quality Assurance, Institute of Quality Science, Poznań University of Economics and Business, Niepodległości 10, 61-875 Poznań, Poland; daniela.gwiazdowska@ue.poznan.pl (D.G.); krzysztof.jus@ue.poznan.pl (K.J.); katarzyna.marchwinska@ue.poznan.pl (K.M.); szymon.frk@gmail.com (S.F.); 2 Department of Chemistry, Poznań University of Life Sciences, Wojska Polskiego 75, 60-625 Poznań, Poland; pascaline.uwineza@up.poznan.pl; 3 Department of Food Safety and Chemical Analysis, Waclaw Dąbrowski Institute of Agricultural and Food Biotechnology-State Research Institute, Rakowiecka 36, 02-532 Warsaw, Poland; dominik.popowski@ibprs.pl (D.P.); daria.padewska@ibprs.pl (D.P.); marek.roszko@ibprs.pl (M.R.); marcin.bryla@ibprs.pl (M.B.); 4 Natural Products and Food Research and Analysis—Pharmaceutical Technology, Faculty of Pharmacy, University of Antwerp, Universiteitplein 1, 2610 Wilrijk, Belgium; 5 Department of Technology and Instrumental Analysis, Institute of Quality Science, Poznań University of Economics and Business, Niepodległości 10, 61-875 Poznań, Poland; katarzyna.pawlak-lemanska@ue.poznan.pl; 6 Research Centre for Registration of Agrochemicals, Institute of Plant Protection-National Research, Władysława Węgorka 20, 60-318 Poznań, Poland; r.gwiazdowski@iorpib.poznan.pl

**Keywords:** phytochemical characterization, supercritical fluid extraction, biological activity, CO_2_ extracts, oregano, antimicrobial and antibiofilm activity

## Abstract

Sustainable management of agri-food product safety presents a major challenge requiring extensive action to ensure food safety and consumer health. The pursuit of environmentally friendly solutions that will constitute an alternative to the chemical compounds commonly used in agriculture and the food industries is one of the most important problems. One solution is plant extracts containing various biologically active compounds and exhibiting antimicrobial activity. This study aims to determine the biological activity of extracts obtained from *Origanum vulgare* L. (leaves) by supercritical CO_2_ (SC-CO_2_) extraction using different reaction conditions and compositions. In vitro studies revealed antimicrobial activity against selected bacteria (including *Salmonella* Enteritidis, *Listeria monocytogenes*, and *Staphylococcus aureus*) and fungi (*Fusarium* spp.), depending mainly on the microorganism species; however, extraction conditions also influenced these properties. The microscopic observations established by optical and fluorescence microscopy showed the changes in the fungal cell’s viability and morphology. There was no observed significant release of intracellular material as stated based on ICP-MS analysis of sodium and potassium concentration. Antibiofilm properties of extract obtained by extraction at 40 °C were also demonstrated against *S. aureus*, *P. aeruginosa*, and *L. monocytogenes*, with stronger properties observed against Gram-positive bacteria. Phytochemical characterization of the extracts was determined using a liquid chromatography system with an orbitrap mass spectrometer (LC/MS), identifying, i.e., phenolic acids: protocatechuic, hydroxybenzoic, caffeic, and rosmarinic; flavonoids: luteolin, naringenin, and kaempferol; and terpenoids: oleanolic and ursolic acids.

## 1. Introduction

Sustainable management of the safety of agri-food products is a major challenge today, requiring diverse and wide-ranging actions to ensure food safety and consumer health. This involves the need to apply solutions that limit the development of pathogenic microorganisms and their transfer to subsequent stages of the food chain. Taking action is required at the stage of plant cultivation in the field, where one of the biggest problems is plant infestations by pathogenic fungi responsible for more than 70% of plant diseases that threaten food security [1]. Moreover, many fungal pathogens, such as *Fusarium* spp., also produce mycotoxins that pose a risk to human and animal health [2,3]. In turn, at the stages of food processing, the problem is microorganisms responsible for food spoilage, pathogenic bacteria such as *Salmonella* sp., *Listeria monocytogenes*, and others, posing a direct threat to the consumer’s health. A particular problem is the formation of biofilms, whose multi-layered, complex structure protects microorganisms against regular cleaning procedures and allows them to survive in the environment. This affects the increased microbiological load of the food processing environment and the final food product quality, leading to spoilage and reduced shelf life, as well as an increased risk of spreading infectious diseases from food [4,5].

The most common solution to limit the spread of undesirable microorganisms is to use substances that inhibit their growth or destroy cells, such as fungicides used during cultivation or disinfectants in the food industry. However, due to their nature, these substances pose a high environmental burden [6,7,8,9]. Therefore, one of the most urgent and important problems is the search for environmentally friendly solutions that will be an alternative to the chemicals commonly used in agriculture and the food industry. A promising alternative to the widely used chemicals is the application of plant extracts containing various biologically active compounds and exhibiting antimicrobial activity, with the possibility of their use at different stages of the food chain. Literature data indicate that numerous medicinal plants rich in alkaloids, flavonoids, and polyphenolic compounds possess antiseptic, antimicrobial, and disinfectant properties without toxic environmental effects [10]. For example, it was noted that adding plant ingredients such as tea tree oil [11] or coffee leaf extract [12] to hand sanitizers or disinfectant sprays effectively inhibited the virus during the COVID-19 pandemic. There are also many reports demonstrating the antifungal effect of different essential oils (EOs) or plant extracts obtained from various plants such as eucalyptus, oregano, clove, cinnamon, lemongrass, and many others, significantly inhibiting the growth of pathogenic fungi, including *Fusarium* species [13,14,15].

*Origanum vulgare* L., known as “oregano”, is a herbaceous species from the *Lamiaceae* family, often used in traditional medicine to treat, among others, diseases associated with inflammation, asthma, indigestion, abdominal pain, bronchitis, cough, and diarrhea [16,17]. There are several groups of plants called “oregano”, including Turkish oregano (*Origanum onites*), Spanish oregano (*Coridohymus capitatu*), Greek oregano (*Origanum vulgare*), and Mexican oregano (*Lippia graveolens*). The best-studied species is Greek oregano (*Origanum vulgare* L.), for which many studies indicate its antibacterial, antioxidant, antifungal, and anti-inflammatory activity. It is primarily attributed to its high content of bioactive compounds such as phenolics, flavonoids, terpenoids, and tannins [18,19,20]. For example, apigenin, a bioflavonoid present in oregano, in addition to its antioxidant properties, is also described as having beneficial effects on the human immune system, blood sugar levels, and several cancers [21]. Luteolin and kaempferol, phytonutrients also observed in oregano, have been studied for their antioxidant, anticancer, and anti-inflammatory properties [22,23,24].

The broad biological activity of oregano aroused great interest in the increasingly widespread use of this plant in various industries and the possibilities of using modern and environmentally friendly extraction technologies. Considering that the extraction step is of the greatest importance for obtaining compounds of interest, the selection and optimization of the extraction method is crucial to obtain the valuable, desired compounds. Methods such as microwave extraction (ME), ultrasonic-assisted extraction (UAE), and supercritical fluid extraction (SFE) are increasingly used [25]. Compared to traditional extraction methods, such as maceration or distillation, their main advantage is the replacement of organic solvents with water, which allows the extraction of bioactive compounds, thus limiting the release of harmful compounds into the environment. Another advantage is the sequential extraction configuration, which consists of reusing solid residues until they are exhausted, allowing for maximum recovery of bioactive substances. Supercritical carbon dioxide (SC-CO_2_) extraction is most commonly used in SFE because it is non-flammable and cheap, and its volatility at atmospheric pressure means that after decompression the extracts are free of solvents. Since for SC-CO_2_ extraction a non-polar solvent is used, a polar modifier (e.g., ethanol, methanol) is often added to enhance the extraction of polar substances. SFE extraction of compounds is used in many areas, including lipids, bioactive substances, essential oils, antioxidants, caffeine, and coloring agents [26,27,28,29].

Considering the above data, the present work aimed to investigate the biological activity and composition of *O. vulgare* extracts obtained via supercritical dioxide extraction with methanol as the co-solvent under differential extraction conditions. The novelty of the presented research is the comprehensive approach to the characterization of the oregano extracts obtained by extraction in supercritical CO_2_, including a broad assessment of biological activity along with composition.

## 2. Results

### 2.1. Antifungal Activity of O. vulgare Extracts

The conducted research showed that the tested oregano SC-CO_2_ extracts, obtained using different temperatures (40 °C—extract abbreviated as OR40, 50 °C—extract abbreviated as OR50, and 60 °C—extract abbreviated as OR60), demonstrated antagonistic activity towards selected fungi of the genus *Fusarium* (Table 1 and Figures S1–S5 in Supplementary Materials). The lowest sensitivity to SC-CO_2_ extracts was observed for *F. culmorum* and *F. poae*, against which MIC (Minimum Inhibition Concentration) and MFC (Minimum Fungicidal Concentration) values of 15.0 mg/mL were recorded for all extract variants. The stronger activity of the tested extracts was observed against *F. graminearum* and *F. avenaceum*, against which MIC and MFC values were at the level of 7.5 mg/mL, regardless of the extraction conditions. Differences in antagonistic activity between the tested variants of oregano extracts were observed only against *F. eguiseti*, where for the OR60 extract, the MIC and MFC values were 1.90 mg/mL, while for the OR40 and OR50 extracts, they amounted to 7.5 mg/mL. It can therefore be concluded that the fungistatic activity of SC-CO_2_ oregano extracts depended mainly on the fungus species, while the extraction conditions generally did not affect the MIC and MFC values.

**Figure 1:**
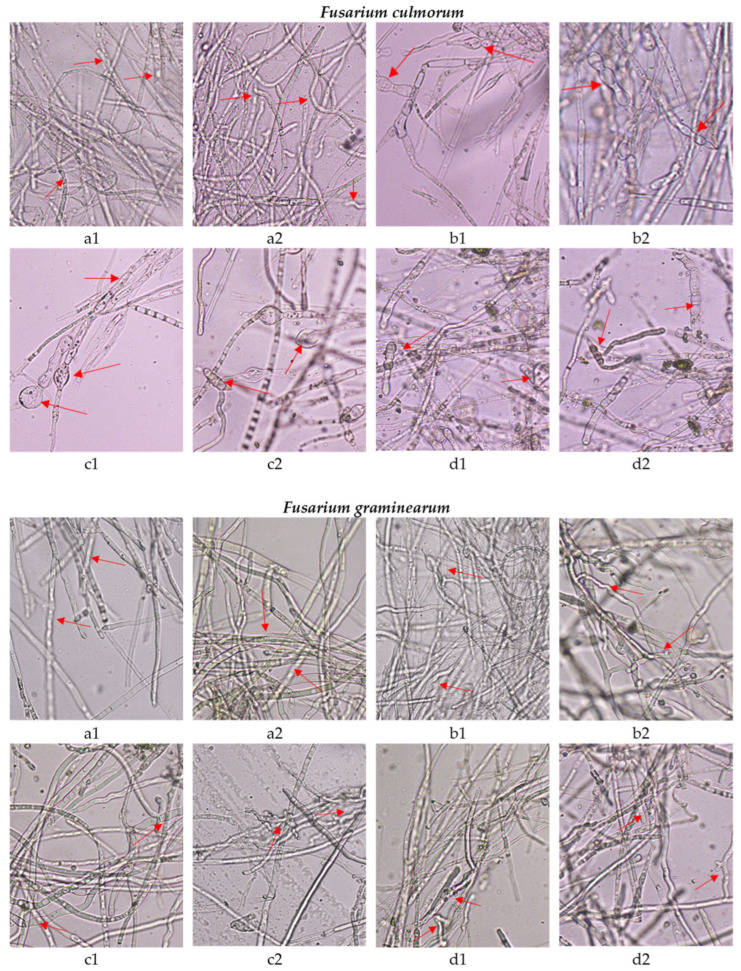
Morphology of *Fusarium* hyphae under optical microscope (magnification 400×): (**a1**,**a2**) untreated, after 1 h and 2 h of incubation; (**b1**,**b2**) treated with OR40 after 1 h and 2 h of incubation; (**c1**,**c2**) treated with OR50 after 1 h and 2 h of incubation; and (**d1**,**d2**) treated with OR60 after 1 h and 2 h of incubation. Scale: 20 µm. Red arrows indicate on the disintegration of the structures inside the fungal hyphae, and its morphological changes.

**Figure 2:**
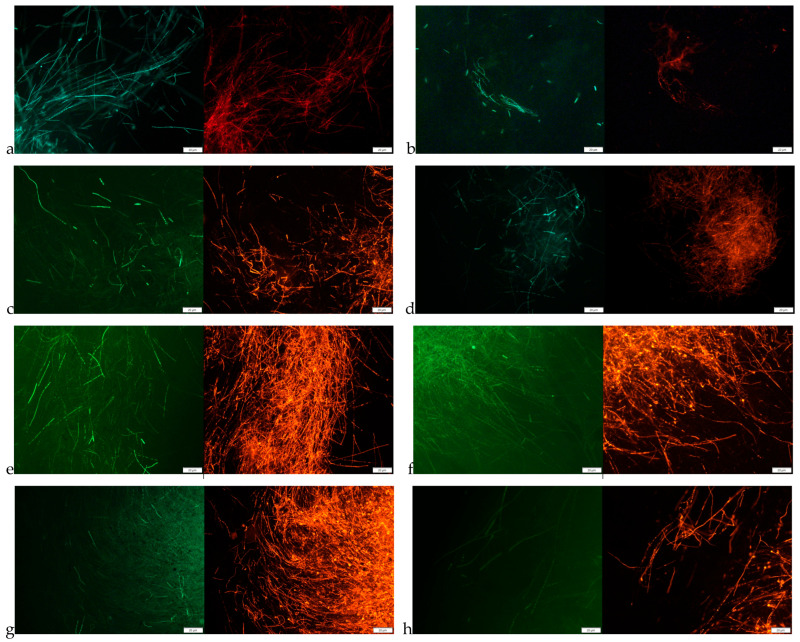
Morphology of *F. culmorum* hyphae under a fluorescence microscope (magnification 400×). (**a**,**b**) Untreated control, stained with FDA (green) and PI (red) after 1 h (**a**) and 2 h (**b**) of incubation; (**c**,**d**) samples treated with OR40 stained with FDA (green) and PI (red) after 1 h (**c**) or 2 h (**d**) of incubation; (**e**,**f**) samples treated with OR50 stained with FDA (green) and PI (red) after 1 h (**e**) or 2 h (**f**) of incubation; and (**g**,**h**) samples treated with OR60 stained with FDA (green) and PI (red) after 1 h (**g**) or 2 h (**h**) of incubation. Scale: 20 µm.

**Figure 3:**
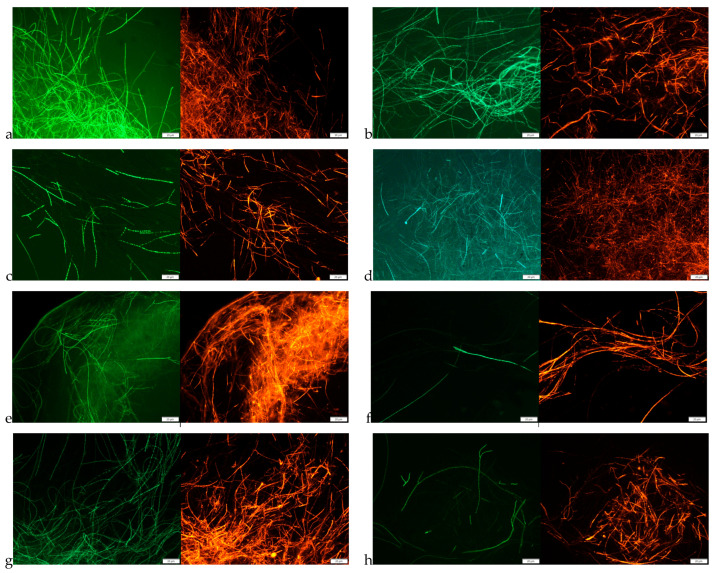
Morphology of *F. graminearum* hyphae under a fluorescence microscope (magnification 400×). (**a**,**b**) Untreated control, stained with FDA (green) and PI (red) after 1 h (**a**) and 2 h (**b**) of incubation; (**c**,**d**) samples treated with OR40 stained with FDA (green) and PI (red) after 1 h (**c**) or 2 h (**d**) of incubation; (**e**,**f**) samples treated with OR50 stained with FDA (green) and PI (red) after 1 h (**e**) or 2 h (**f**) of incubation; and (**g**,**h**) samples treated with OR60 stained with FDA (green) and PI (red) after 1 h (**g**) or 2 h (**h**) of incubation. Scale: 20 µm.

The fungistatic activity of tested oregano SC-CO_2_ extracts was also confirmed in fluorescence microscopy studies by analyzing the effect of the extracts on mature mycelium. Microscopic analyses were performed for the *F. culmorum* and *F. graminearum* by incubating the mycelium with the SC-CO_2_ extracts for 1 and 2 h. The results are presented in Figure 1. Based on the obtained microscopic images, it can be stated that the oregano SC-CO_2_ extracts affect both the structure of the hyphae and their viability. The microscopic images showed a clear disintegration of the structures inside the fungal hyphae, and morphological changes, as thickening and swelling were detected. A characteristic swelling was observed in the hyphae of *F. culmorum*, whereas the cells of *F. graminearum* were mainly distorted by bending. Additionally, in the images obtained from fluorescence microscopy (Figure 2 and Figure 3) after the use of fluorescein diacetate (FDA) and propidium iodide (PI) dyes, an increasing share of dead hyphae (red) was noted after incubation of the mycelium in the presence of the tested oregano SC-CO_2_ extracts compared to the control samples (a larger number of live hyphae—green). Similarly to the previous analyses, no major differences were observed in the fungistatic activity of the oregano SC-CO_2_ extracts depending on the extraction conditions. The sensitivity of the fungi was mainly related to the incubation time in the presence of the tested *O. vulgare* L. extracts.

### 2.2. Release of Cell Constituents

**Figure 4:**
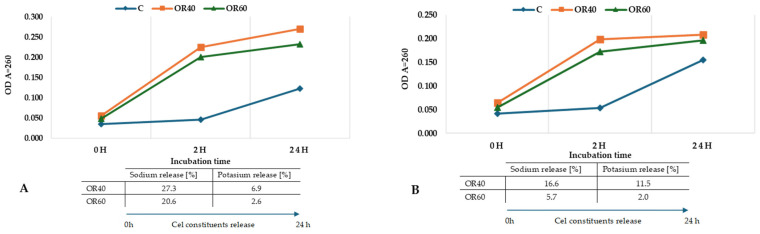
Effect of oregano SC-CO_2_ extracts on the cellular constituents’ efflux of *Fusarium* fungi: (**A**)—*F. graminearum*; (**B**)—*F. culmorum*; C—control; OR40—oregano SC-CO_2_ extract obtained using 40 °C temperature; OR60—oregano SC-CO_2_ extract obtained using 60 °C temperature.

To determine the preliminary mechanism of antifungal action, an attempt was made to assess membrane integrity by measuring optical density (OD) at 260 nm and determining the efflux of selected intracellular components, sodium, and potassium by the inductively coupled plasma ionization mass spectrometer method (ICP-MS). This study was carried out for two extracts, OR40 and OR60 since the extract obtained at 50 °C did not differ in terms of biological activity from OR40. As the results in Figure 4 indicate, the absorbance increased slightly during the 24 h of incubation. Similarly, the concentration of sodium and potassium increased from 5.7 to 27.3% of Na and 2.0 to 11.5% of K, depending on the type of extract and fungal species.

### 2.3. Antibacterial and Antibiofilm Activity of O. vulgare Extracts

#### 2.3.1. Antibacterial Properties

The antibacterial properties of the tested oregano SC-CO_2_ extracts were determined against eight indicator microorganisms from the group of Gram-positive and Gram-negative bacteria. Based on the obtained results, it can be stated that the *O. vulgare* SC-CO_2_ extracts, in comparison to fungistatic activity, showed higher antagonistic activity against selected indicator bacteria, where MIC and MBC (Minimum Bactericidal Concentration) values were at the level of 0.25 to 1.0 mg/mL (Table 2). A slightly higher sensitivity to the influence of SC-CO_2_ extracts was noted towards Gram-positive bacteria, the growth of which was inhibited by 90–100% at the concentration of extracts of 0.25 mg/mL (OR40 and OR50) or 0.5 mg/mL (OR60). Only against *E. faecalis*, the OR60 SC-CO_2_ extract showed higher antagonistic activity (MIC of 0.25 mg/mL) than towards other microorganisms. Analysis of the antibacterial properties of the tested SC-CO_2_ *O. vulgare* extracts against Gram-negative bacteria showed that regardless of the extraction conditions, the MIC values were 0.5 mg/mL. Higher MIC/MBC values (1.0 mg/mL) were observed only for the OR60 extract against *C. jejuni* and *P. aeruginosa*.

It should be emphasized that the differences in the antibacterial activity of individual extracts expressed by MIC values are not large, and generally, only for a few microorganisms do the MIC and MBC values differ slightly depending on the extract. This is probably related to the composition of the extracts, described in point 2.4. As the composition analysis showed, the extracts did not differ substantially in terms of quality, except for several compounds for which the signal intensity (in positive mode) was significantly higher in the extract obtained at 60 °C (Table 3).

#### 2.3.2. Antibiofilm Activity of Oregano SC-CO_2_ Extracts

**Figure 5:**
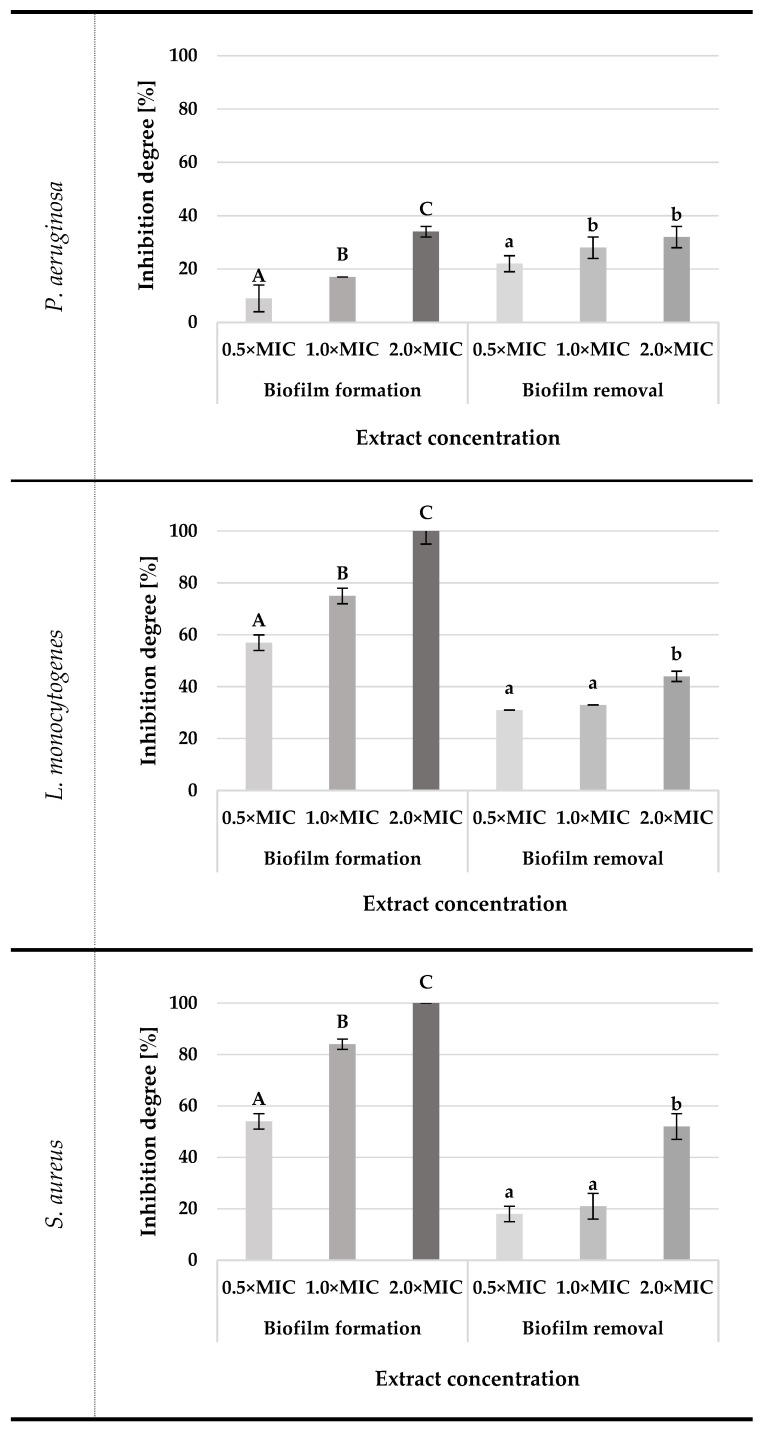
Effect of *Origanum vulgare* L. SC-CO_2_ extracts OR40 on biofilm formation and removal: averages with different letters (A–C) for biofilm formation and (a,b) for biofilm removal for each microorganism are significantly different at the *p* < 0.05.

Determining the activity of the tested SC-CO_2_ oregano extracts in preventing the formation and removal of mature bacterial biofilm was important considering the potential use of these compounds in the food industry. This study was carried out against three bacteria: *P. aeruginosa*, *L. monocytogenes*, and *S. aureus*, which have a high ability to form biofilm in the production environment [30]. Since no major differences in antibacterial activity were observed between the individual variants of the tested SC-CO_2_ extracts, only the OR40 extract (in three concentrations) was used in the experiment. Based on the obtained results, presented in Figure 5, it can be concluded that the type of indicator microorganism and the concentration of the OR40 extract had a key impact on the formation and removal of bacterial biofilm. The degree of inhibition of the biofilm formation process depended significantly on the extract concentration used. Regardless of the indicator microorganism, the inhibition degree increased with increasing extract concentration. The efficacy of inhibiting the biofilm formation process varied depending on the microorganism. The weakest effect was noted for *P. aeruginosa*, where at a concentration of 2×MIC, the degree of inhibition of biofilm formation was 34%. Much better efficacy was obtained for *L. monocytogenes* and *S. aureus*. OR40 extract at a concentration of 2×MIC completely inhibited the biofilm formation process by these two microorganisms. In contrast, at a concentration of 0.25 mg/mL (MIC) and 0.5×MIC, biofilm formation was inhibited by 75% and 57% for *L. monocytogenes* and 84% and 54% for *S. aureus*, respectively.

OR40 SC-CO_2_ extract demonstrated the ability to remove mature biofilm with efficiency ranging from 18% to 52%, depending on the SC-CO_2_ extract concentration and the type of tested microorganism. The degree of *P. aeruginosa* biofilm removal was significantly higher at the extract concentration of 0.5 MIC and 1.0xMIC, while no significant differences were observed at the lowest concentration. No significant differences were found in the effect of the extract on the removal of mature biofilm of *S. aureus* and *L. monocytogenes*, but the effect of the highest concentration was significantly higher. The best results were observed for the 2.0×MIC extract concentration in the case of *S. aureus* and *L. monocytogenes* biofilms, with the removal of 52% and 44%, respectively. The obtained results differed significantly from the results obtained for lower extract concentrations for these bacterial strain biofilms. For both tested lower extract concentrations—0.5×MIC and 1.0×MIC—no significant statistical differences were found as the biofilm removal efficiency was 31% and 33% for *L. monocytogenes* and 18% and 21% for *S. aureus*, respectively. The lowest sensitivity to the OR40 SC-CO_2_ extract was demonstrated by the *P. aeruginosa* biofilm, which was removed by only 32% at the extract concentration of 2.0×MIC. No significant difference concerning the extract concentration of 1.0×MIC was observed as the biofilm removal equaled 28%.

### 2.4. Phytochemical Characterization of O. vulgare SC-CO_2_ Extracts

**Figure 6:**
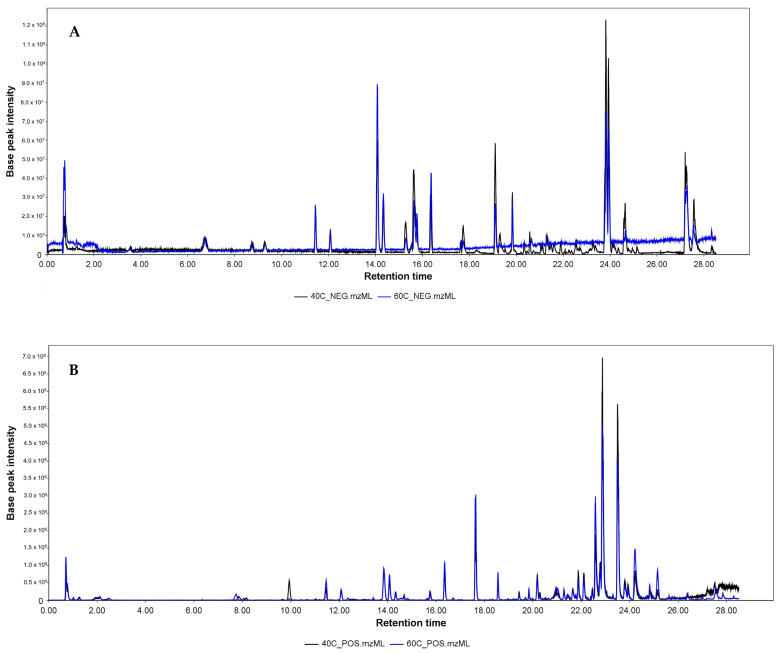
The LC–MS analysis of the extracts obtained at 40 °C (in black) and 60 °C (in blue). Base peak chromatograms acquired in negative (**A**) and positive (**B**) ionization modes.

The liquid chromatography–mass spectrometry (LC–MS) analysis of *Origanum vulgare* extracts obtained at 40 and 60 °C in negative and positive ionization modes shows in most cases no difference between the composition of the extracts (Figure 6). Only in the case of a few compounds (**10**, **18**, **21**, **33**, and **34**) was a statistically significant difference (*p* < 0.05) demonstrated for the extract obtained at 60 °C compared to the one obtained at 40 °C (Table 3).

The phytochemical analysis of the extracts was performed in both ionizations separately to provide data suitable for semiquantitative comparison and highlight the differences in particular constituents’ extraction yields in particular SC-CO_2_ conditions.

The results (Table 3) revealed the presence of well-described constituents of oregano herb, like phenolic acids: protocatechuic (**2**), hydroxybenzoic (**3**), caffeic (**4**), and rosmarinic (**12**); flavonoids: luteolin (**17**), naringenin (**19**), and kaempferol (**25**); and terpenoids: oleanolic (**35**) and ursolic (**36**) acids. Additionally, multiple unidentified molecular features were observed during the analysis of the extract. Number **7** was identified as a monoterpenoid—loliolide, previously described in *Origanum vulgare* [31]. Number **8** might be tentatively identified as protocatechuic acid derivatives based on the presence of *m*/*z* 153.020 and 109.030 ions on the fragmentation spectrum. Number **16** was tentatively identified as eriodictyol, a flavonoid previously described in other plants from the *Lamiaceae* family [32]. Based on fragmentation, number 11 might be a structural isomer of number **16** (as similar fragmentary ions, *m*/*z* 269.050, 151.006, 125.027, and 109.031, were detected). Numbers **28** and **30**, although not identified, are probably structural isomers, sharing similar fragment ions at *m*/*z* 344.089 and 326.079 (**28**)/326.066 (**30**). A similar relationship occurs in the case of numbers **31** and **32**, which have close signals originating from pseudomolecular ions (at *m*/*z* 293.21205 (**31**) and 293.21210 (**32**) in negative mode, and m/z 277.21425 (**31**) and 277.21470 (**32**) in positive mode), as well as in fragmentation spectra. Comprehensive fragmentary ion lists were included in Table 3 to enable convenient comparison and reference, especially in the case of unidentified constituents.

## 3. Discussion

In the era of sustainable development goals and increasing concern for the environment and human and animal welfare, more and more attention is being paid to finding environmentally friendly solutions in various areas, including antimicrobial substances, which are used intensively in plant production and food processing. The present work demonstrates the possibilities for the CO_2_ extracts from *O. vulgare* used as antimicrobial or antibiofilm compounds at different stages of the food chain. It is worth emphasizing that, although more and more data can be found on CO_2_ extracts, the number of comprehensive studies examining their broad biological activity in combination with the composition and different extraction conditions is still limited. Furthermore, the authors test different extraction conditions, so the results are not always comparable.

Obtained, in presented research, data indicate that the lower extraction temperatures (40 and 50 °C) of oregano SC-CO_2_ extracts resulted in higher antibacterial properties towards *S. aureus*, *M. luteus*, *E. faecalis*, and *C. jejuni*, while this effect was not observed for the other indicator bacteria. No such relationship was observed concerning the indicator fungi. Due to the popularity of oregano and its properties, it is a plant used quite often in research; however, the results regarding antimicrobial properties reported in the literature differ from each other and those obtained in this study. Santoyo et al. (2006) [33] also used SFE to obtain *O. vulgare* L. extracts under different process parameters determined for their antimicrobial properties. The authors observed antibacterial activity of all extracted fractions towards *S. aureus*, *B. subtilis*, *E. coli*, *P. aeruginosa* as well as antifungal properties towards *Aspergillus niger* and *Candida albicans*. However, the most active oregano fraction was obtained with 7% ethanol at 150 bar and 40 °C. The most susceptible tested microorganism was *C. albicans*, and the least was *A. niger*. Karakaya et al. (2011) [34] examined the antioxidant and antibacterial properties of SC-CO_2_ extracts, obtained at 40 °C temperature and 105 bar pressure from oregano (*Origanum vulgare* ssp. *hirtum*). However, all SFE extracts did not affect the growth of the tested bacterial strains: *L. monocytogenes*, *S. aureus*, *E. coli*, and *S. typhimurium*. The authors considered that the low amount of oxygenated compounds in SFE extract was the cause of the lack of antibacterial properties. The reason is that the phenolic components of extracts pose the strongest antimicrobial activity, followed by aldehydes, ketones, and alcohols. Nevertheless, it should be noted that the content of polyphenolic compounds does not fully reflect the profile of compounds with antimicrobial activity of the plant, which is affected by the presence of other substances such as alkaloids, coumarins, terpenes, aldehydes, carotenoids, carvacrol, or sulfur-containing compounds [35]. Other authors also confirm the antimicrobial activity of SFE extracts from various plants, often emphasizing the dependence of activity on the extraction conditions. Therefore, it can be stated that lower SC-CO_2_ extraction temperature may result in higher antimicrobial properties of the extracts; however, their activity is related to the extraction techniques used, different parameters of the process, the concentration of active substances, and finally the tested microorganism strains.

Literature data confirm the ability of plant extracts to inhibit bacterial biofilm formation [36,37]. It is worth mentioning that bacteria have different potentials for biofilm formation. This ability is mainly determined by the amount of EPS produced, i.e., compounds responsible for the attachment of bacterial cells to the colonized surface [38]. The bacteria *B. cereus*, *C. jejuni*, *E. coli*, *L. monocytogenes*, *S*. Enterica, *S. aureus*, and *P. aeruginosa* are most often used as model microorganisms in biofilm studies due to their proven ability to form such structures on various surfaces, including in the food industry [30]. The results concerning the removal of mature biofilm by OR40 SC-CO_2_ extract indicated moderate activity, which may be related to the specific structure of bacterial biofilms, responsible for their resistance to various types of cleaning and disinfecting agents. Due to the different methodological approaches of various authors, to compare the results, the percentage inhibition of biofilm at the tested concentration of 1×MIC was guided, regardless of the MIC value determined by the author of the study. Bazargani and Rohloff (2016) [39], found that the tested oregano essential oils MIC concentration (4 mg/mL) inhibited *S. aureus* biofilm formation by more than 50%. In comparison, the results obtained by Vidaković et al. (2023) [40] indicate that oregano extract at MIC = 0.09–0.72 µL/mL inhibited *L. monocytogenes* biofilm formation in a range of 42% to 62% depending on the medium variant and the concentration of extracts. Ben Abdallah, Lagha, and Gaber (2020) [41] demonstrated the ability of oregano oil to destroy mature *S. aureus* biofilm at 60–76% when using concentrations above 3.125 mg/mL. The efficiency of removing mature biofilm was lower than the efficiency of inhibiting biofilm formation. The weaker biofilm removal efficiency compared to its inhibition efficiency at the formation stage is directly related to the complexity of the biofilm’s multi-layered, adhesive structure, whose matrix protects the cells living inside by impeding the penetration of active substances. Even though plant substances have been proven to effectively destabilize biofilm structures due to their ability to diffuse into the interior of the polysaccharide matrix, resulting in high intrinsic antimicrobial activity [42], the results indicate that mature biofilm structures of Gram-negative bacteria are highly resistant to the action of selected plant extracts.

Many studies indicate that plant extracts can destroy the integrity of the cell wall; therefore, in this study, the effect of the extracts obtained on cell morphology and cell wall continuity was assessed using the example of the fungi *F. culmorum* and *F. graminearum*. This study showed that the extracts strongly affect cell morphology and also lead to the destruction of the fungal cell wall, as evidenced by the change in cell color observed under a fluorescence microscope and the efflux of intracellular substances. However, such strong changes as observed by some authors who treated were not noted. Helal et al. [43] observed the effect of *Cymbopogon citratus* essential oil on the cell membrane disruption and leakage of intracellular constituents. Similarly, Zhang et al. [44] described the disruption of *Botrytis cinerea* with morphological changes in cells, including deformation and deterioration of the hyphae and conidia.

In addition to the biological activity of the tested extracts, the composition was determined. Oregano is found almost all over the world, most associated with the Mediterranean area; therefore some reports characterize the composition of extracts depending on the type of extraction. Essential oils rich in components such as carvacrol and thymol, as well as p-cymene, y-terpinene, caryophyllene, spathulenol, and germacrene-D, are responsible for oregano’s characteristic aroma and biological properties, and their percentages can vary significantly depending on the plant’s origin and growing conditions [45]. Busatta et al. (2017) [46] tested the chemical profiles of oregano extracts obtained by supercritical CO_2_, at 40 °C and 200 atm, finding *cis*-sabinene hydrate being the major acquired compound. It was conducted that the number of oxygenated monoterpenes in the oregano extract obtained by SC-CO_2_ totaled 58% of the identified compounds. In the present works, many well-described constituents of oregano herb, belonging to phenolic acids, flavonoids, and terpenoids; however, some unidentified molecular features were also observed.

## 4. Materials and Methods

### 4.1. Plant Material

Dried, ground *O. vulgare* L. herbs were bought from a Polish producer of high-quality herbal products called Dary Natury, located in Podlaskie Voivodeship (53°4′10.98 latitude and 22°58′2.87 longitude).

### 4.2. Origanum vulgare Sample Extraction

The extract of *O. vulgare* was obtained by a supercritical CO_2_ extraction technique/procedure, following the methodology described by Uwineza et al. (2021) [47]. The dried and grounded oregano herbs in the amount of 5 g were inserted in an extraction vessel of volume 25 mL and maintained in an oven at different temperatures (40, 50, and 60 °C) and constant pressure (250 bar). The flow rate of CO_2_ was set to 4 mL/min, and 1 mL/min of pure methanol (99.5% purity), which was used as a co-solvent. The extraction process started automatically after the established conditions were reached and conducted for 180 min in every experimental run, which consisted of 1st dynamic time—45 min, static time—15 min, and 2nd dynamic time—120 min. *O. vulgare* extracts were finally collected in flasks located in a fraction collection module and, for further studies, stored at −20 °C.

### 4.3. Chemicals

The acetonitrile and water of MS grade used to prepare LC–MS phases were purchased from Witko (Łódź, Poland). Phase additives: ammonium formate (≥97%) and formic acid (98–100%) (LC–MS grade) were supplied by Chem-Lab (Zedelgem, Belgium). Methanol was purchased from POCh (Gliwice, Poland). Fluorescein diacetate and propidium iodide were bought from Sigma-Aldrich (Steinheim, Germany). Carbon dioxide (SFE grade) was purchased from Air Products Ltd. (Poznań, Poland). Microbiological media were obtained from BioMaxima (Lublin, Poland) and A&A Biotechnology (Gdańsk, Poland). All chemicals used for the studies were of analytical grade.

### 4.4. Antimicrobial Properties of O. vulgare Extracts

#### 4.4.1. Antifungal Activity—Minimal Inhibitory Concentration (MIC) and Minimal Fungicidal Concentration (MFC) Assay

The fungistatic activity of the tested extracts was determined against five filamentous fungi. The species of the genus *Fusarium* (*F. graminearum* KZF 1, *F. culmorum* KZF 5, *F. avenaceum* KZF 3, *F. equiseti* KZF 6, and *F. poae* KZF 181) were obtained from the collection of the Research Centre for Registration of Agrochemicals, Institute of Plant Protection, National Research Institute in Poznań, Poland. The tested filamentous fungi were cultivated in Petri dishes (55 mm diameter) on Potato Dextrose Agar (PDA) at 25 °C for 5–10 days. The MIC and MFC of the tested *O. vulgare* extracts were determined using the microdilution method according to Gwiazdowska et al. (2022) [48] with some modifications. First, the suspensions of hyphae and conidia were prepared in sterile PDB by mixing harvested mycelium from mature cultures with medium to achieve a final cell concentration of 10^6^ cells/mL, determined with a hemocytometer. Next, twofold dilutions of the extracts were prepared in 96-well microtiter plates in PDB with a final concentration established in the range of 0.08–10 mg/mL. The final concentration of methanol in control samples was established in the range of 0.2–25% in proportion to its content in the samples. Next, 100 µL of the microorganism solutions were added to each well. The microtiter plates were incubated under aerobic conditions at 25 °C ± 2 °C for 5–10 days. Culture media containing *O. vulgare* extracts without fungal inoculum were used as negative controls, whereas fungal cultures excluding extracts were used as positive controls. MIC values for filamentous fungi were first determined through a visual assessment of the fungal growth on the plate. The MFC value was determined via spot inoculation of 10 μL of microbial culture. All tests were performed in triplicate.

#### 4.4.2. Antibacterial Activity—Minimal Inhibitory Concentration (MIC) and Minimal Bactericidal Concentration (MBC) Assay

Antibacterial activity of *O. vulgare* extracts was determined towards four Gram-positive bacteria—*Micrococcus luteus* ATCC 10240, *Staphylococcus aureus* ATCC 33862, *Bacillus subtilis* ATCC 11774, *Enterococcus faecalis* ATCC 19433, and *Listeria monocytogenes* ATCC 19115—as well as four Gram-negative bacteria—*Escherichia coli* ATCC 8739, *Pseudomonas aeruginosa* ATCC 9027, *Salmonella enterica* ser. Enteritidis ATCC 13076, and *Campylobacter jejuni* ATCC 33291. All bacterial strains were purchased from the American Type Culture Collection (ATCC) and cultivated on liquid or agar media: nutrient broth/agar (NB/NA) for *S. aureus*, *B. subtilis*, *E. coli*, and *P. aeruginosa*, trypticasein soy broth/agar (TSB/TSA) for *M. luteus*; and brain heart infusion broth/agar (BHI) for *E. faecalis*, *L. monocytogenes*, *S. Enteritidis*, and *C. jejuni*, under 30 °C for *M. luteus* and 37 °C for the remaining bacteria. Before the experiment, all bacteria were transferred to the appropriate agar media and cultivated for 24 h. Next, inocula in Mueller–Hinton broth (MHB) with optical density adjusted to 0.5 McFarland standard were prepared. The microdilution method according to Gwiazdowska et al. (2022) [46] with some modifications was used to determine the MIC and MBC values of the tested *O. vulgare* extracts. Twofold dilutions of the extracts in MHB were prepared in 96-well microtiter plates with the final concentration established in the range of 0.04–5 mg/mL. Next, 100 µL of the microorganism solutions were added to each well. The plates were covered and incubated for 24 h at 30 °C or 37 °C, depending on the microorganism. Culture media containing *O. vulgare* extracts without bacterial inoculum were used as negative controls, whereas bacterial cultures without extracts were used as positive controls. After incubation, the optical density of the bacterial samples was determined at a 600 nm wavelength using the BioTek Epoch 2 microplate reader. The MIC value was defined as the lowest concentration of extract that exhibited at least 90% growth inhibition, and the MBC value was determined as 100% inhibition based on spectrophotometric measurements using BioTek Epoch 2.

### 4.5. Release of Cell Constituents

The release of cell constituents was determined according to the method described by Paul et al. [49] and Zhang et al. [44] with some modifications. The mycelia of *F. culmorum* and *F. graminearum* harvested from PDA were suspended in 10 mL of sterile water with the addition of OR40 and OR60 at a concentration equal to 1×MIC and incubated at a temperature of 24 °C for 24 h. After 2 h and 24 h, samples were collected and centrifuged for 2 min at 14,000× *g*. The supernatant was used for determining the absorbance at 260 nm.

Cell membrane damage was assessed by determining sodium and potassium concentration in releasing intracellular material directly after extract application (0 h) and after 24 h incubation in PBS by the inductively coupled plasma ionization mass spectrometer method (ICP-MS) following ISO 17294-2:2023 [50]. The detection limit for the elements determined was 1 µg/mL. Immediately before measurement, samples were centrifuged (14,000 rpm for 20 min) to obtain a supernatant without cellular residues.

### 4.6. Antibiofilm Activity of O. vulgare Extract

The antibiofilm properties of *O. vulgare* extract were examined in two ways: as an agent that causes and inhibits the process of biofilm formation by *P. aeruginosa*, *L. monocytogenes*, and *S. aureus* and once formed to remove mature biofilm according to the method described by Gwiazdowska et al. (2022) [46]. The extract obtained by extraction at 40 °C was chosen for the experiment due to similar results of antimicrobial activity of three tested extracts. Biofilm formation was carried out according to the modified Somrani (2020) [51] method. In the first step, standardized bacteria cultures of 10^6^ CFU/mL were prepared in the nutrient broth and added into each well in amounts of 60 µL. To determine the effect of the tested extracts on the ability to form bacterial biofilms, 60 µL of extract at concentrations equal to 0.5 MIC, 1 MIC, and 2 MIC were introduced into each well of flat-bottom 96-well microtiter plates together with inoculum. The prepared plates were incubated for 24 h at 37 °C, after which the suspension was removed from the wells and rinsed three times with sterile water to remove non-adherent cells and the residual medium. Then, the microtitre plates were air-dried for 2 h, and the biofilm was stained with crystal violet. The efficacy of removing the mature biofilm with the tested extract was determined after the biofilm formation by introducing 60 µL of the bacterial inoculum into each well and incubating for 24 h at 37 °C. After incubation, the biofilm was washed with water and air-dried. The biofilm prepared in this way was exposed to the extract by washing three times with 125 µL of the extract at concentrations equal to 0.5 MIC, 1 MIC, and 2 MIC. After 15 min at room temperature (25 °C ± 1 °C), the extract was removed, and the plates were washed with water, air-dried for 2 h, and stained with crystal violet. Methanol was used as a negative control.

In both cases, biofilm biomass was quantified using the modified crystal violet method developed by O’Toole [52]. To the formed, dried biofilm, 125 µL of a 0.1% crystal violet solution was added, incubated for 15 min, and then the plates were washed with water and dried overnight. Next, 125 µL of 30% acetic acid was introduced into wells and left for 10-15 min at room temperature (25 °C ± 1 °C). The solution from each well was transferred to a new microtiter plate, and the optical density was analyzed at a 550 nm wavelength using a BioTek Epoch 2 microplate reader. As a blank, 30% acetic acid in water was used. All experiments were conducted in triplicate parallel repetitions. The obtained results were expressed as a percentage of inhibition of biofilm formation.

### 4.7. Morphology Observations

The microscopic observations were carried out for selected fungi: *F. culmorum* and *F. graminearum*. After cultivation in PDB (Potato Dextrose Broth) for 5 days at 25 °C, the mycelia of tested fungi were harvested from the surface of the medium and suspended in 1 mL of sterile water with the addition of *O. vulgare* extracts at a concentration equal to 1×MIC for 1 h and 2 h of incubation. Samples were transferred on glass slides and photographed with a light microscope (Olympus BX53, Olympus Corporation, Tokyo, Japan) at 400× magnification. Fungal samples treated with sterile water were used as control samples. Each assay was repeated in triplicate.

### 4.8. Staining and Fluorescence Microscopy

The fluorescent probes, fluorescein diacetate (FDA), and propidium iodide (PI) (Sigma Aldrich, Steinheim, Germany), were applied to assess the viability of fungi treated with *O. vulgare* extracts. The staining procedure was based on the method described by Wang et al. 2022 [53] and Gwiazdowska et al. 2022 [54]. A stock solution of FDA (1 mg/mL) in acetone and a stock solution of PI (1 mg/mL) in distilled water were prepared and stored in the refrigerator. The tested strains, *F. culmorum* and *F. graminearum*, were prepared and incubated as described in Section 4.8. Next, 25 μL of FDA and 5 μL of PI were added to the samples, which were incubated with fluorescent dyes for 30 min at 30 °C. The staining solution was removed by centrifugation, and the cell pellet was transferred on glass slides and examined under the fluorescence microscope Olympus BX53 (Olympus Corporation, Tokyo, Japan) equipped with specific wavelength filters set (FDA excitation/emission: 485/530; PI excitation/emission: 538/617). Fungal samples treated with sterile water were used as control samples.

### 4.9. Phytochemical Characterization of the O. vulgare SC-CO_2_ Extracts

For the analytical screening, Cortecs 100 mm × 2.1 mm × 1.6 µm C18 column (Waters, Milford, MA, USA) was used. The mobile phase (A) was water with 0.1% formic acid and 5 mM ammonium formate, and the mobile phase (B) was acetonitrile:water 80:20 with 0.1% formic acid and 5 mM ammonium formate. The gradient program was as follows: 0–3.5 min 0% B, 3.5–16 min 0–26% B, 16–26.5 min 26–100% B, 26.5–28.5 min 100% B. The flow was 0.3 mL/min. The column oven temperature was 45 °C. Samples were filtered using 0.22 µm nylon syringe filters before injection. The injection volume was 2 µL. The analyses were performed using a Vanquish UHPLC system hyphenated with an Exploris 120 orbitrap mass spectrometer (Thermo Fisher Scientific, Waltham, MA, USA). The blank sample (50% MeOH_aq_) analysis was performed to prepare the exclusion list and increase the MS2 data output from the experimental samples. The extracts obtained at 40 and 60 °C were analyzed in both ionization modes separately (in three repetitions) in MS1-only mode and DDA acquisition mode with the exclusion list implemented in the method. The ESI parameters were as follows: capillary voltage 3.5 kV and 2 kV in positive and negative mode, respectively; sheath gas flow 48 AU; auxiliary gas 11 AU; sweep gas 2 AU; ion transfer tube temperature 320 °C; and vaporizer temperature 350 °C. The acquisition parameters were as follows: MS1 resolution 120,000, MS2 resolution 15,000, scan range *m*/*z* 100–1500, profile data acquisition, normalized HCD collision energy at 30%. The data were processed using CompoundDiscoverer 3.3 (Thermo Scientific, Austin, TX, USA). The preset metabolomics with identification workflow with changes was used with GNPS and mzCloud library search included. The MZmine 4.3 was used for visualization and data inspection.

### 4.10. Statistical Analysis

The results are presented as the arithmetic mean (±standard deviation) from three parallel replicates. In addition, selected results were estimated by one-way analysis of variance (ANOVA) using Tukey’s test with a significance level of *p* < 0.05. For all statistical analyses, the Microsoft Excel^®^ and IBM SPSS Statistics 29 (PS IMAGO PRO 9.0) programs were used.

## 5. Conclusions

The development of research on environmentally friendly antimicrobial substances is increasingly directing attention to modern extraction methods such as supercritical CO_2_ extractions. The results obtained in this study showed the significant potential of oregano extracts obtained by SC-CO_2_ extraction against a wide spectrum of microorganisms, both bacteria and fungi. Despite slight differences in the range of biological activity, the most promising results were obtained for the extract obtained at 40 °C. This provides great opportunities for their use at different stages of the food chain, both during plant cultivation and in the food industry. Accordingly, *O. vulgare* L. SC-CO_2_ extracts show promising potential for inclusion into food products and sustainable agriculture in which an antimicrobial additive is needed as a safe alternative to synthetic antimicrobials. Moreover, the SFE process involves the use of an environmentally clean technology. It is worth emphasizing that both the method of obtaining extracts and the scope of the characterization introduce an element of scientific novelty, because, to the best of the authors’ knowledge, there are not many similar studies.

## Figures and Tables

**Table 1:** Fungistatic activity (MIC/MFC) of oregano SC-CO_2_ extracts against selected *Fusarium* spp.

Fungi	MIC and MFC of Oregano SC-CO_2_ Extracts [mg/mL] *			
		OR40	OR50	OR60
*F. graminearum* KZF 1	MIC	7.5	7.5	7.5
	MFC	7.5	7.5	7.5
*F. culmorum* KZF 5	MIC	15.0	15.0	15.0
	MFC	15.0	15.0	15.0
*F. poae* KZF 181	MIC	15.0	15.0	15.0
	MFC	15.0	15.0	15.0
*F. equiseti* KZF 6	MIC	7.5	7.5	1.90
	MFC	7.5	7.5	1.90
*F. avenaceum* KZF 3	MIC	7.5	7.5	7.5
	MFC	7.5	7.5	7.5

* OR40—oregano SC-CO_2_ extract obtained using 40 °C temperature; OR50—oregano SC-CO_2_ extract obtained using 50 °C temperature; OR60—oregano SC-CO_2_ extract obtained using 60 °C temperature.

**Table 2:** Antibacterial activity (MIC/MBC) of oregano SC-CO_2_ extracts against selected Gram-positive and Gram-negative bacteria.

Indicator Bacteria	MIC and MBC of Oregano SC-CO_2_ Extracts [mg/mL] *			
		OR 40	OR 50	OR 60
Gram-positive bacteria				
*S. aureus*	MIC	0.25	0.25	0.5
	MBC	0.25	0.25	0.5
*M. luteus*	MIC	0.25	0.25	0.5
	MBC	0.25	0.25	0.5
*E. faecalis*	MIC	0.25	0.25	0.25
	MBC	0.25	0.25	0.5
*L. monocytogenes*	MIC	0.25	0.25	0.5
	MBC	0.5	0.5	0.5
Gram-negative bacteria				
*E. coli*	MIC	0.5	0.5	0.5
	MBC	0.5	0.5	0.5
*P. aeruginosa*	MIC	0.5	0.5	1.0
	MBC	1.0	1.0	1.0
*C. jejuni*	MIC	0.5	0.5	1.0
	MBC	1.0	1.0	1.0
*S. enteritidis*	MIC	0.5	0.5	0.5
	MBC	0.5	0.5	0.5

* OR40—oregano SC-CO_2_ extract obtained using 40 °C temperature; OR50—oregano SC-CO_2_ extract obtained using 50 °C temperature; OR60—oregano SC-CO_2_ extract obtained using 60 °C temperature.

**Table 3:** Composition of *Origanum vulgare* extracts obtained at 40 and 60 °C analyzed in negative and positive ionization modes.

No.	RT	MS1 (−)	MS2 (−)	MS1 (+)	MS2 (+)	ID	Ratio (60/40) *
1	0.78	191.05594	173.059; 127.036			Quinic acid	
2	4.71	153.01936	109.031			Protocatechuic acid	
3	7.50	137.02446	94.038			Hydroxybenzoic acid	
4	9.62	179.03492	135.048			Caffeic acid	
5	11.44	387.16547	207.120; 163.126; 119.031; 101.031; 89.030; 59.012	411.16172	249.11		
6	12.08	225.11310	147.084; 97.068; 59.015	227.12547	209.117; 191.107; 167.107; 163.112; 149.096; 131.085; 121.101; 85.065		
7	13.82			197.11514	179.107; 161.096; 151.112; 135.117; 133.101; 107.085	Loliolide	
8	14.07	437.10829	153.020; 121.030; 109.030	456.14521	155.034; 123.044		
9	14.33	421.11349	153.031	440.15057	107.049		
10	14.67			453.33827	435.283; 336.252; 326.246; 209.147; 100.115		1.73
11	15.40	287.05600	269.050; 259.066; 243.070; 215.075; 201.059; 199.076; 180.009; 177.059; 151.006; 125.027; 109.031; 83.015				
12	15.75	359.07668	197.049; 179.034; 161.027; 135.047			Rosmarinic acid	
13	15.87	421.11347	137.025	440.15057	139.029; 123.058; 111.717		
14	16.35	451.12406	167.035	470.16063	169.057; 123.048		
15	17.63			679.51129	661.501; 552.448; 452.360; 435.333; 336.228; 326.280; 209.165		
16	17.86	287.05611	269.046; 151.004; 135.045; 125.024; 109.030			Eriodictyol	
17	18.39	285.04055	257.046; 241.051; 217.051; 199.041; 175.040; 151.004; 133.031			Luteolin	
18	18.56			905.69183 ([M+H]+); 453.33863 ([M+2H]+2)	887.668; 778.616; 678.529; 661.502; 562.397; 552.448; 452.359; 435.333; 336.228; 209.165		1.55
19	19.19	271.06116	253.054; 177.022; 165.022; 151.006			Naringenin	
20	19.44			253.17791	235.169; 217.159; 199.149; 189.164; 175.112; 161.096; 157.101; 147.117; 119.086; 99.080		
21	19.84			351.21019	333.217; 281.112		1.27
22	20.18			181.12076	163.112; 135.117; 121.101; 107.085; 95.086		
23	20.98			345.09412	330.073; 315.050; 313.065; 284.068		
24	21.43			291.19341	273.185; 217.159; 157.101; 145.101; 135.081; 131.085; 117.070; 109.065		
25	21.51	285.07680	243.089; 228.962; 151.015; 93.039			Kaempferol	
26	21.64	283.06124	268.038			Acacetin	
27	21.88			361.12530	197.041; 191.067		
28	22.10			359.10964	344.089; 326.079; 298.083		
29	22.87			329.09911	314.047; 296.039		
30	23.50			359.10953	344.089; 329.066; 311.056; 272.760; 150.982		
31	23.80	293.21205	275.202; 211.134; 183.134; 171.103; 121.103	277.21425	259.185; 235.149; 221.164; 149.138; 133.106; 121.106; 107.078; 93.064		
32	23.90	293.21210	275.202; 223.134; 195.139	277.21470	259.206; 221.153; 149.133; 135.117; 121.101; 107.086; 93.070		
33	25.14			532.37494 ([M+NH4]+); 515.36334 ([M+H]+)	435.713; 258.255; 200.741; 100.268		4.23
34	25.16			488.34993 ([M+NH4]+); 471.32343. ([M+H]+)	471.351; 277.216; 233.191; 165.091; 133.086; 121.065; 89.060		3.65
35	27.26	455.35250				Oleanolic acid	
36	27.58	455.35245				Ursolic acid	

RT—retention time; MS1—observed *m*/*z* values; MS2—fragment ions *m*/*z* in negative (−) and positive (+) ionization modes; ID—identified compounds; * signal intensity (positive mode) ratio for the extract obtained at 60 °C to the one obtained at 40 °C (in the case when *p* < 0.05 in the means’ comparison).

## Data Availability

The data presented in this study are available in the article and the Supplementary Materials.

## Supplementary Materials

The following supporting information can be downloaded at https://www.mdpi.com/article/10.3390/molecules29245823/s1, Figure S1. Fungistatic activity of tested oregano SC-CO_2_ extracts against *F. graminearum* (C—control; MeOH—methanol at concentration 25%, 12.5% and 6.25% respectively; OR40, OR50, OR60—oregano SC-CO_2_ extracts); Figure S2. Fungistatic activity of tested oregano SC-CO_2_ extracts against *F. culmorum* (C—control; MeOH—methanol at concentration 25%, 12.5% and 6.25% respectively; OR40, OR50, OR60—oregano SC-CO_2_ extracts); Figure S3. Fungistatic activity of tested oregano SC-CO_2_ extracts against *F. poae* (C—control; MeOH—methanol at concentration 25%, 12.5% and 6.25% respectively; OR40, OR50, OR60—oregano SC-CO_2_ extracts); Figure S4. Fungistatic activity of tested oregano SC-CO_2_ extracts against *F. equiseti* (C—control; MeOH—methanol at concentration 25%, 12.5% and 6.25% respectively; OR40, OR50, OR60—oregano SC-CO_2_ extracts); Figure S5. The fungistatic activity of tested oregano SC-CO_2_ extracts against *F. avenaceum* (C—control; MeOH—methanol at concentration 25%, 12.5%, and 6.25% respectively; OR40, OR50, OR60—oregano SC-CO_2_ extracts).
