# Supplementary material for: Antimicrobial and Antibiofilm Activity of Origanum vulgare Extracts Obtained by Supercritical Fluid Extraction Under Various Extraction Conditions
Source: Molecules. 2024 Dec 10;29(24):5823. doi: 10.3390/molecules29245823 (PMC11676637; doi:10.3390/molecules29245823)
Supplement: Supplementary file 1 [file molecules-29-05823-s001.zip › molecules-3351020-supplementary.pdf]

molecules

Supplementary Material

Antimicrobial and Antibiofilm Activity of *Origanum vulgare* Extracts Obtained by Supercritical Fluid Extraction under Various Extraction Conditions

Daniela Gwiazdowska [1], Agnieszka Waśkiewicz [2,*], Krzysztof Juś [1], Katarzyna Marchwińska [1], Szymon Frąk[1], Dominik Popowski [3,4], Katarzyna Pawlak-Lemańska [5], Pascaline Aimee Uwineza [2], Romuald Gwiazdowski [6], Daria Padewska [3], Marek Roszko [3], and Marcin Bryła [3]

[1] Department of Natural Science and Quality Assurance, Institute of Quality Science, Poznań University of Economics and Business, Niepodległości 10, 61-875 Poznań, Poland; daniela.gwiazdowska@ue.poznan.pl (D.G.), krzysztof.jus@ue.poznan.pl (K.J.) katarzyna.marchwinska@ue.poznan.pl (K.M.), szymon.frk@gmail.com (S.F.)

[2] Department of Chemistry, Poznań University of Life Sciences, Wojska Polskiego 75, 60-625 Poznań, Poland; agnieszka.waskiewicz@up.poznan.pl (A.W.)

[3] Department of Food Safety and Chemical Analysis, Prof. Waclaw Dabrowski Institute of Agricultural and Food Biotechnology-State Research Institute, Rakowiecka 36, 02-532 Warsaw, Poland; dominik.popowski@ibprs.pl (D.Po.); daria.padewska@ibprs.pl (D.Pa.); marek.roszko@ibprs.pl (M.R.); marcin.bryla@ibprs.pl (M.B.)

[4] Natural Products and Food Research and Analysis - Pharmaceutical Technology, Faculty of Pharmacy, University of Antwerp, Universiteitplein 1, Wilrijk, Belgium; dominik.popowski@uantwerpen.be (D.Po.)

[5] Department of Technology and Instrumental Analysis, Institute of Quality Science, Poznań University of Economics and Business, Niepodległości 10, 61-875 Poznań, Poland; katarzyna.pawlak-lemanska@ue.poznan.pl (K.P.-L.)

[6] Research Centre for Registration of Agrochemicals, Institute of Plant Protection-National Research Institute, Władysława Węgorka 20, 60-318 Poznań, Poland; R.Gwiazdowski@iorpib.poznan.pl (R.G.)

* Correspondence: agnieszka.waskiewicz@up.poznan.pl (A.W.)

Citation: To be added by editorial staff during production.

Academic Editor: Firstname Lastname

Received: date
Revised: date
Accepted: date
Published: date

Copyright: © 2023 by the authors. Submitted for possible open access publication under the terms and conditions of the Creative Commons Attribution (CC BY) license (https://creativecommons.org/licenses/by/4.0/).

1. Supplementary Material

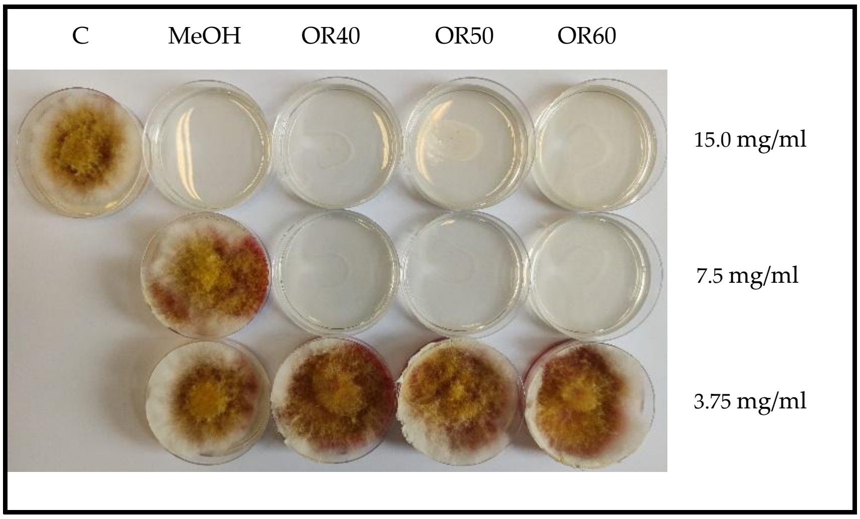

Fig.S1. Fungistatic activity of tested oregano SC-CO_2 extracts against *F. graminearum* (C – control; MeOH – methanol at concentration 25%, 12.5% and 6.25% respectively; OR40, OR50, OR60 – oregano SC-CO_2 extracts)

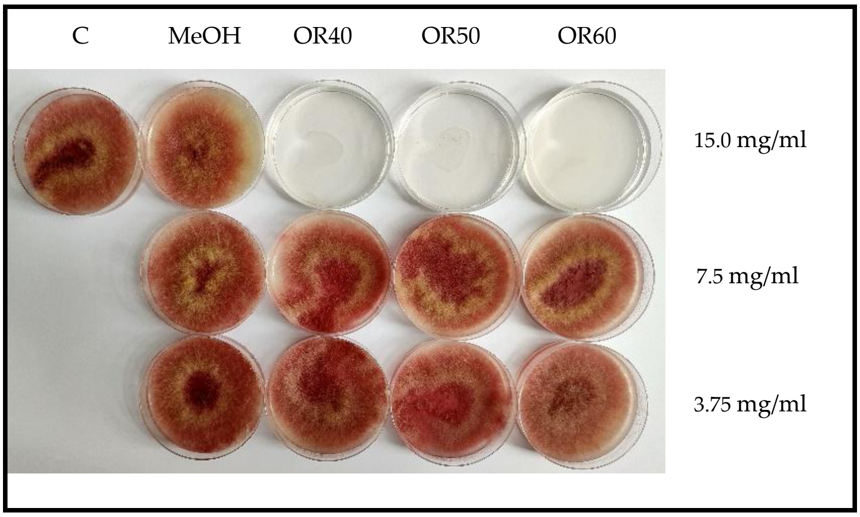

Fig.S2. Fungistatic activity of tested oregano SC-CO_2 extracts against *F. culmorum* (C – control; MeOH – methanol at concentration 25%, 12.5% and 6.25% respectively; OR40, OR50, OR60 – oregano SC-CO_2 extracts)

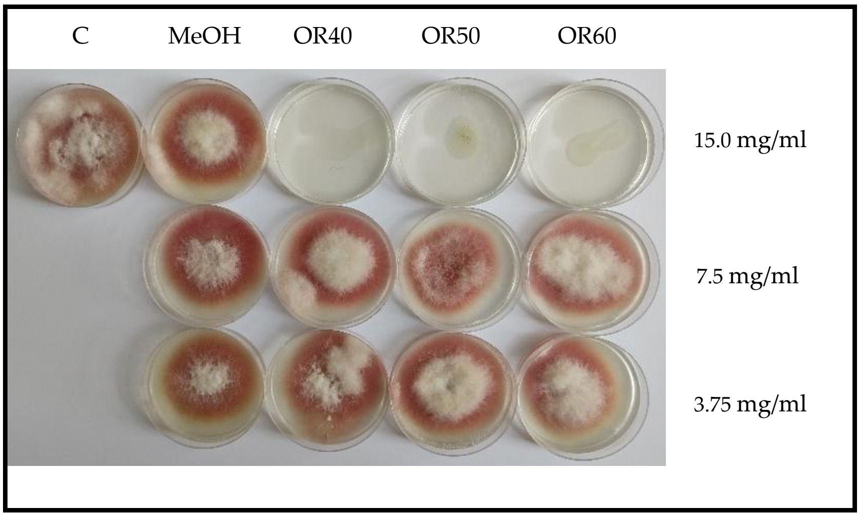

Fig.S3. Fungistatic activity of tested oregano SC-CO_2 extracts against *F. poae* (C – control; MeOH – methanol at concentration 25%, 12.5% and 6.25% respectively; OR40, OR50, OR60 – oregano SC-CO_2 extracts)

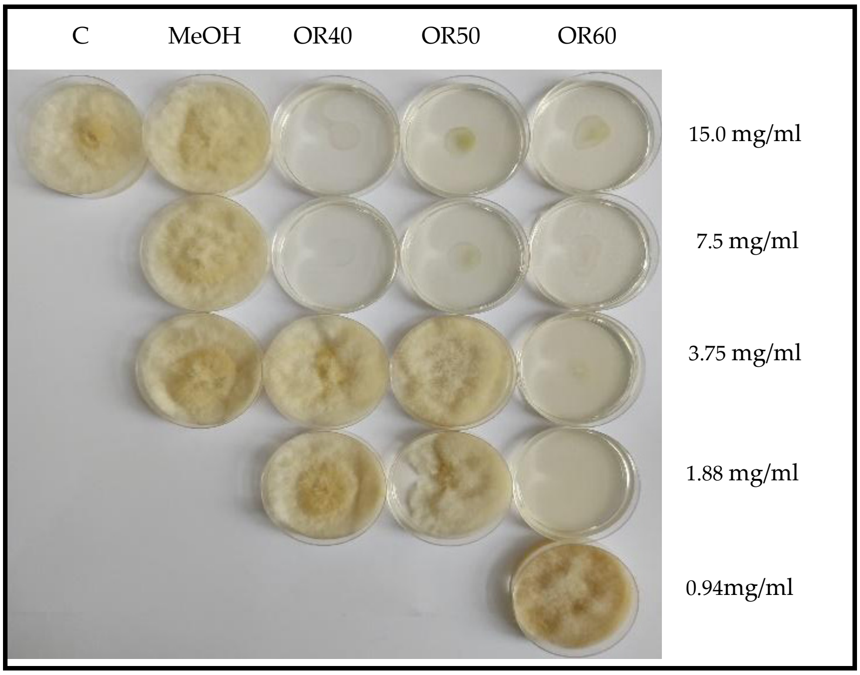

Fig.S4. Fungistatic activity of tested oregano SC-CO_2 extracts against *F. equiseti* (C – control; MeOH – methanol at concentration 25%, 12.5% and 6.25% respectively; OR40, OR50, OR60 – oregano SC-CO_2 extracts)

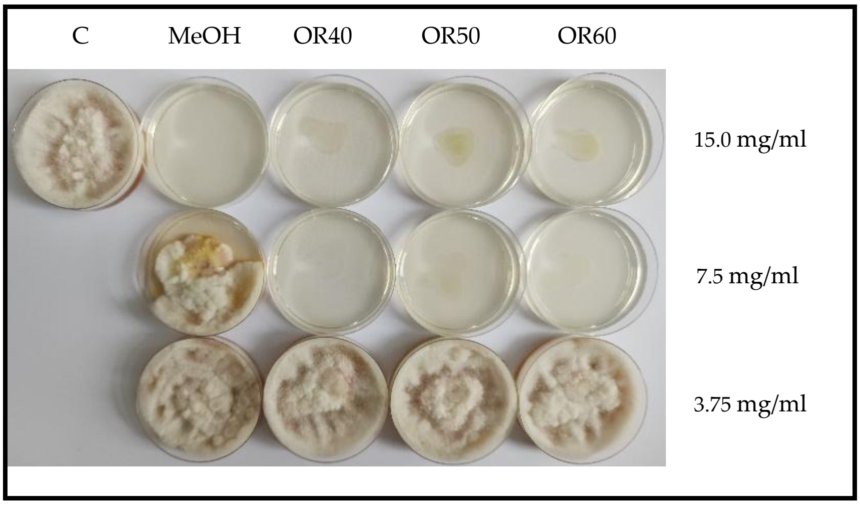

Fig.S5. Fungistatic activity of tested oregano SC-CO_2 extracts against *F. avenaceum* (C – control; MeOH – methanol at concentration 25%, 12.5% and 6.25% respectively; OR40, OR50, OR60 – oregano SC-CO_2 extracts)
